# Teriflunomide-Induced Palmoplantar Pustular Psoriasis: Case Report and Review of the Literature

**DOI:** 10.7759/cureus.42845

**Published:** 2023-08-02

**Authors:** Ezgi Demirel Ozbek, Neslihan Akdogan, Deniz Ates Ozdemir, Nazire Pinar Acar Ozen, Aslı Tuncer

**Affiliations:** 1 Department of Neurology, Hacettepe University, Ankara, TUR; 2 Department of Dermatology, Hacettepe University, Ankara, TUR; 3 Department of Pathology, Hacettepe University, Ankara, TUR

**Keywords:** multiple sclerosis, palmoplantar pustular psoriasis, adverse effects, teriflunamide, immunomodulatory treatments

## Abstract

Teriflunomide is a once-daily oral immunomodulatory disease-modifying treatment for multiple sclerosis (MS). Skin reactions are an infrequent side effect of teriflunomide. Here, we present the case of a 52-year-old female patient with ankylosing spondylitis who was consulted for demyelinating lesions and limb weakness. She was diagnosed with multiple sclerosis and started treatment with teriflunomide. Palmoplantar pustular psoriasis developed after three weeks of treatment initiation. It is a rare side effect related to teriflunomide.

## Introduction

Multiple sclerosis (MS), one of the leading causes of disability in young adults, is an autoimmune disease characterized by inflammation, demyelination, and axonal loss of the central nervous system (CNS). It is typically diagnosed in young adults aged 20 to 30 years and affects women approximately three times more than men. Although previously it was believed to be principally T-cell driven, in the last few decades it has been accepted that B cells, myeloid cells, and an abnormal balance between effector and regulatory cells are also important in the pathophysiology of MS [1]. Since the first disease-modifying therapy (DMT), interferon beta-1b, which was approved by the U.S. Food and Drug Administration (FDA) in 1993 [2], numerous oral, injectable, and intravenous medications have been investigated and added to DMTs.

Teriflunomide (Aubagio®; Sanofi Genzyme, Framingham, MA, USA) is a once-daily oral immunomodulatory DMT for the treatment of MS. In animal studies and clinical trials, it has been shown to reduce the proliferation of activated T and B lymphocytes via inhibition of dihydro-orotate dehydrogenase, which is a mitochondrial enzyme responsible for de novo pyrimidine synthesis [3]. Hair thinning, diarrhea, nausea, headache, urinary tract infection, and an increase in hepatic enzymes are the most common side effects of teriflunomide [4]. Teriflunomide-related skin reactions are scarce. There are only a few case reports regarding cutaneous adverse effects of teriflunomide, such as eczema, rash, bullous pemphigoid [5], and toxic epidermal necrolysis [6]. Palmoplantar pustular psoriasis induced by teriflunomide was first described in 2019 [7], and only three cases have been reported so far.

## Case presentation

A 52-year-old female patient was referred to our neurology clinic in May 2022 for an evaluation of right leg weakness for the last four years. She was diagnosed with ankylosing spondylitis in January 2021 and used adalimumab for one year with no benefit. Therefore, adalimumab was switched to hydroxychloroquine by the rheumatology department, and the patient was referred to our department due to weakness in her right leg. Her spinal MRI showed one vertebral-length demyelinating plaque located in the C3, C6, T5, and T10 segments. Demyelinating plaques were found in the pons, corpus callosum, and periventricular white matter. She was evaluated for the possible etiologies of primary or secondary (anti-tumor necrosis factor (TNF)-induced) demyelination of the central nervous system. At the time of her admission to our department in May 2022, she was complaining about urinary urgency, weakness in her right leg, and numbness in her lower extremities and back. Her adalimumab treatment was discontinued. Her muscle strength was 4/5 in her right leg, and her tandem gait was abnormal in her neurologic examination. In her lumbar puncture, the immunoglobulin G (IgG) index was 1.04, and oligoclonal bands were positive. Chronic demyelinating plaques were also seen in her previous cranial MRI from 2019.

Taking her symptoms, neurological findings, MRI, and cerebrospinal fluid (CSF) results into consideration, the patient was diagnosed with multiple sclerosis. Treatment with teriflunomide oral, 14 mg daily, was initiated in July 2022. Three weeks after initiating teriflunomide, the patient was admitted for a denuding skin rash on her extremities. She was referred to our dermatology outpatient clinic.

**Figure 1:**
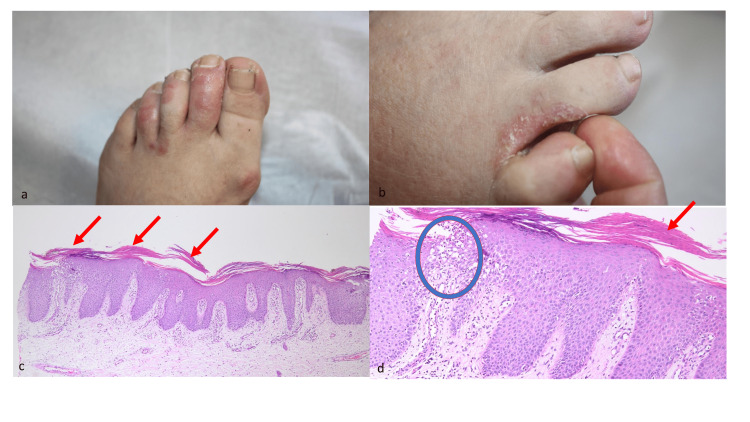
Imaging and histopathological features of the lesions 1a and 1b: erythematous desquamative plaques on the dorsum of the second, third, and fourth toes, as well as between the fourth and fifth toes, associated with scaling and fissuring. 1c: hyperkeratosis, parakeratosis in the stratum corneum (arrow), regular acanthosis in the epidermis, thinning/loss of the granular layer (40x, H&E). 1d: neutrophils, parakeratosis, orthokeratosis (arrow) in the stratum corneum, and intraepidermal neutrophils are present in the epidermis (circle) (100x, H&E). H&E: hematoxylin and eosin stain

The dermatologic evaluation revealed that there were erythematous desquamative plaques on the dorsum of the second, third, and fourth toes, as well as between the fourth and fifth toes, associated with scaling and fissuring (Figures 1a-1b).

Since the patient received topical antifungal treatment without any benefit, a 4-mm punch biopsy was taken from her left foot with a provisional diagnosis of palmoplantar psoriasis. Hematoxylin and eosin sections showed a psoriasiform reaction pattern characterized by hyper-parakeratosis, orthokeratosis, and neutrophils in the stratum corneum, regular acanthosis in the epidermis, and thinning of the granular layer (Figure 1c). In the papillary dermis, there was a dilated capillary network, some of which were congested (Figure 1d). The findings were compatible with the psoriasiform reaction (Figures 1c-1d), and therefore she was diagnosed with teriflunomide-induced palmoplantar psoriasis.

Although there are some case reports of adalimumab-induced palmoplantar psoriasis in patients with ankylosing spondylitis [8], adalimumab was not blamed as a causative agent in this patient since she stopped using adalimumab long before switching to teriflunomide therapy. The patient's eruptions regressed after seven months of cessation and elimination of teriflunomide. During this time, calcipotriol/betamethasone ointment was added to her medical treatment, which contributed to the improvement.

## Discussion

Psoriasis is a chronic, proliferative, and inflammatory skin disease characterized by scaly papules and plaques that can involve any part of the body. Having an autoimmune nature, MS and psoriasis share similarities in their immunopathogenesis [9]. Teriflunomide's potential link to psoriasis is not yet fully understood, but it could involve a paradoxical reactivation of inflammatory pathways related to psoriasis due to the blockade of pyrimidine synthesis in susceptible individuals, maintaining a relevant level of innate and/or adaptive immunity through an aberrant feedback mechanism [10].

Palmoplantar psoriasis (PP) is a chronic variant of psoriasis, accounting for 3%-4% of all psoriasis cases and affecting mostly palms and soles [11]. Palmoplantar psoriasis can be triggered by stress, smoking, irritants, friction, and trauma in genetically susceptible individuals [12]. It can also be triggered by certain medications, including lithium, beta (β)-blockers, nonsteroidal anti-inflammatory drugs, antibiotics, angiotensin-converting enzyme inhibitors, and anti-tumor necrosis factor drugs [13]. To the best of our knowledge, including our case, there have been four cases of palmoplantar psoriasis induced by teriflunomide [7, 13, 14]. To our knowledge, this is the first case of teriflunomide-induced palmoplantar psoriasis in a patient known to have ankylosing spondylitis and prior use of adalimumab.

Clinical and demographic features of patients with teriflunomide-induced palmoplantar psoriasis are listed in Table 1.

**Table 1:** Clinical and demographic features of patients with teriflunomide-induced palmoplantar psoriasis PP: palmoplantar psoriasis

Author	Age	Gender	Medical history	History of any dermatological disease	MS treatment before TRF	Appearance of lesions after TRF exposure	Successful dermatological treatment for lesions	Therapy after PP
Demirel Ozbek et al., 2023	52	F	Ankylosing spondylitis	No	None	3 weeks	Calcipotriol and topical corticosteroid	Ocrelizumab
Negretto et al., 2019 [7]	52	F	No important features	No	Interferon beta1a 44 mcg	1 month	Topical corticosteroid	Dimethyl fumarate
Agirgol et al., 2019 [14]	54	F	Hypothyroidism and fibrocystic breast disease	No	Dimethyl fumarate	2 months	Topical corticosteroid	Not reported
Couper et al., 2022 [13]	52	M	No important features	No	None	3 days	Combination of calcipotriol and acitretin	Teriflunomide

All of the patients were between the ages of 50 and 55. The female-to-male ratio was 3:1. Only one patient (25%) had a rheumatological disease and a history of previous anti-TNF use. None of the patients had psoriasis or any other dermatoses before teriflunomide therapy. Lesions appeared between three days and two months after the initiation of teriflunomide. Three patients (75%) required drug discontinuation. Three patients responded well to topical corticosteroid therapy; however, one patient was steroid-resistant but responsive to acitretin. No relapses occurred after teriflunomide cessation and, subsequently, psoriasis treatment in these cases.

## Conclusions

Herein, we present a patient with MS concomitant with ankylosing spondylitis who experienced palmoplantar psoriasis following teriflunomide use. Psoriasis triggered by teriflunomide is not common. Neurologists should be aware of the dermatological side effects of teriflunomide. Regression of lesions after cessation of teriflunomide is an important clue leading to the diagnosis. A multidisciplinary approach is essential in suspected cases.
